# Lifestyle intervention reduces risk score for cardiovascular mortality in company employees with pre-diabetes or diabetes mellitus – A secondary analysis of the PreFord randomized controlled trial with 3 years of follow-up

**DOI:** 10.3389/fendo.2023.1106334

**Published:** 2023-02-23

**Authors:** Christian Brinkmann, Hannah Hof, Detlef-Bernd Gysan, Christian Albus, Stefanie Millentrup, Birna Bjarnason-Wehrens, Joachim Latsch, Gerd Herold, Karl Wegscheider, Christian Heming, Melchior Seyfarth, Hans-Georg Predel

**Affiliations:** ^1^ Institute of Cardiovascular Research and Sport Medicine, Department of Preventive and Rehabilitative Sport Medicine, German Sport University Cologne, Cologne, Germany; ^2^ Department of Fitness and Health, IST University of Applied Sciences, Düsseldorf, Germany; ^3^ Medical Center for Cardiology, Angiology, Pneumology and Rehabilitation Medicine, Cologne, Germany; ^4^ Department of Psychosomatics and Psychotherapy, University of Cologne, Cologne, Germany; ^5^ Fresenius University of Applied Sciences, Cologne, Germany; ^6^ Health Service of the Ford Motor Company GmbH, Cologne, Germany; ^7^ Institute of Medical Biometry and Epidemiology, University of Hamburg-Eppendorf, Hamburg, Germany; ^8^ HELIOS University Hospital Wuppertal, Wuppertal, Germany; ^9^ Witten/Herdecke University, Witten, Germany

**Keywords:** exercise, nutrition, cardiovascular risk assessment, employees, diabetes

## Abstract

**Aim:**

To evaluate the effects of a multimodal intervention (including exercise training, psychosocial interventions, nutrition coaching, smoking cessation program, medical care) on the health and long-term cardiovascular disease (CVD) mortality risk of company employees with pre-diabetes or diabetes mellitus (DM) at high CVD risk.

**Methods:**

In the PreFord study, German company employees (n=4196) participated in a free-of-charge CVD mortality risk screening at their workplace. Based on their European Society of Cardiology – Systematic Coronary Risk Evaluation score (ESC-SCORE), they were subdivided into three risk groups. High-risk patients (ESC-SCORE≥5%) were randomly assigned to a 15-week lifestyle intervention or usual care control group. Data from patients with pre-DM/DM were analyzed intention-to-treat (ITT: n=110 versus n=96) and per protocol (PP: n=60 versus n=52).

**Results:**

Body mass index, glycated hemoglobin, total cholesterol, low-density lipoprotein, triglyceride levels as well as systolic and diastolic blood pressure improved through the intervention (ITT, PP: p<0.001). The ESC-SCORE markedly decreased from pre- to post-intervention (ITT, PP: p<0.001). ESC-SCORE changes from baseline differed significantly between the groups, with the intervention group achieving more favorable results in all follow-up visits 6, 12, 24 and 36 months later (at each time point: ITT: p<0.001; PP: p ≤ 0.010).

**Conclusion:**

The study demonstrates the feasibility of attracting employees with pre-DM/DM at high CVD mortality risk to participate in a multimodal lifestyle program following a free CVD mortality risk screening at their workplace. The lifestyle intervention used in the PreFord study shows high potential for improving health of company employees with pre-DM/DM in the long term. ISRCTN23536103.

## Graphical Abstract

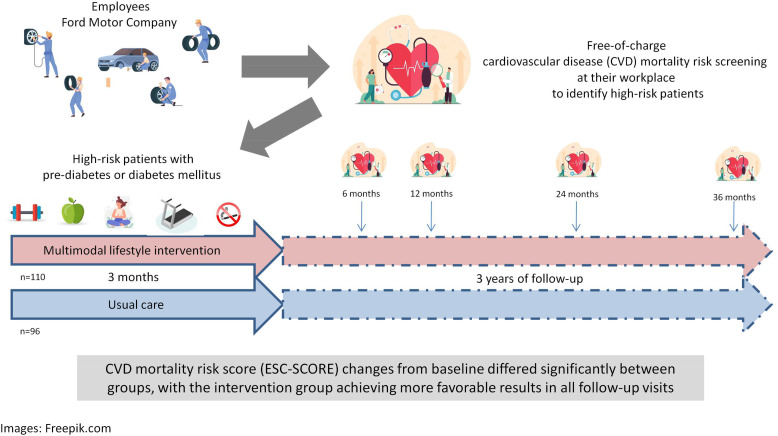

## 1 Introduction

Cardiovascular diseases (CVDs) are the leading cause of premature death (1, 2). Thus, reducing the incidence of CVDs is of high public health importance. A meta-analysis from observational studies has shown that a healthy lifestyle can reduce the risk of developing CVDs by up to 66% (3). Preventive measures aimed at lifestyle changes can therefore be helpful to reduce individual mortality risk. In the PreFord study (4), German company employees of the Ford Motor Company (n=4196) participated in a free-of-charge CVD mortality risk screening at their workplace. The participants were then subdivided into three risk groups based on their risk factors, quantified by the European Society of Cardiology – Systematic Coronary Risk Evaluation score (ESC-SCORE), which is an established metric to estimate the risk of fatal cardiovascular events with a high accuracy for Germans and other Europeans (5, 6). Employees with a high risk score (ESC-SCORE ≥ 5%) were randomly assigned to a multimodal lifestyle intervention group (receiving exercise training, psychosocial interventions, nutrition coaching, smoking cessation program, medical care) or to a usual care group (receiving medical care only).

Large-scale observational studies show that patients with diabetes mellitus (DM) have a drastically increased risk of cardiovascular events and CVD mortality (7–9). As lifestyle changes can help reduce cardiovascular risk, patients with pre-DM and DM should optimize their lifestyle as early as possible. Unfortunately, these patients are often very difficult to motivate for lifestyle changes; moreover, there might be several psycho-social barriers (10). When they participate in an intervention program, achieving sustainable effects is usually challenging, due to low program adherence and high drop-out rates (11).

This secondary analysis of the PreFord study data explores the direct effects of the study’s 15-week multimodal lifestyle intervention on the ESC-SCORE and other health-related variables in the pre-DM/DM subgroup. Long-term effects on the individual cardiovascular risks and the program’s efficiency for patients with pre-DM/DM are discussed, considering that aggressive programs for lifestyle changes are urgently needed to account for an increasing incidence and prevalence of DM (12, 13).

## 2 Methods

### 2.1 PreFord study

#### 2.1.1 Study design

The PreFord trial was designed as a randomized controlled, multicenter clinical study. The study design has already been described in detail (4). The study protocol in line with good clinical practice has been approved by the Ethics Committee of the University of Cologne (ref: 03-217) and the Ethics Committee of the North Rhine Medical Association (Ärztekammer Nordrhein, ref: 2004079). Subjects gave their written informed consent prior to the start of the study.

#### 2.1.2 Subjects

Employees of the Ford Motor Company Germany (>15.000) were invited to participate in a free-of-charge cardiovascular medical check-up (T0) and to determine their ESC-SCORE which reflects personal risk of cardiovascular events. The score was calculated by an independent statistics institution (Institute of Medical Statistics, Informatics and Epidemiology, University of Cologne). Age, blood pressure, smoking habits and total cholesterol values were recorded for risk assessment. Inclusion criteria were defined as follows: an ESC-SCORE ≥ 5% (high-risk group) and the ability to exercise. Exclusion criteria were defined as follows: exercise-limiting diseases, history of cardiovascular disease, cancer, pregnancy or severe mental disorders.

#### 2.1.3 Lifestyle intervention

Subjects were randomly assigned to the intervention (INT) group or the usual care control (CON) group by block randomization 1:1. The computer-generated random list was provided by the Clinical Trial Center Cologne. Study personnel assigned participants to the INT or CON group according to this random list. The 15-week multimodal lifestyle intervention (**Table 1**) was supervised by professional health care specialists (medical doctors, exercise physiologists, psychologists, and nutritional coaches). The intervention was performed in small groups twice a week for 2.5-3 hours per session in two rehabilitation centers in Cologne, Germany. Further details about the program are available in the publication of Gysan et al. (4). All employees who participated in the intervention program were examined immediately after the intervention (T1). The CON group participants received usual care from their general practitioners.

**Table 1:** Lifestyle intervention.

Components	Hours planned	Subgroup data: Actual time spent (percentage of planned hours)
Aerobic endurance and resistance training	37.00	90.0%
Nutrition coaching Information/Education in Mediterranean-style diet and practical training in preparing a meal	11.00	73.1%
LifeSkills according to Williams and Williams	13.50	74.5%
Progressive relaxation training	6.00	90.7%
Smoking cessation program	0.45	11.1% (5 persons)
Medical care with guideline-based pharmacotherapy	4.75	90.7%
Information/Education Healthy lifestyle management	8.00	121.9%

#### 2.1.4 Follow-up

All company employees who participated in the study, in either the INT or ON group, were invited for follow-up medical check-ups 6 (T2), 12 (T3), 24 (T4) and 36 (T5) months after start of the study. The study ended after completion of the last follow-up.

### 2.2 Secondary data analysis

#### 2.2.1 Subjects

**Figure 1:**
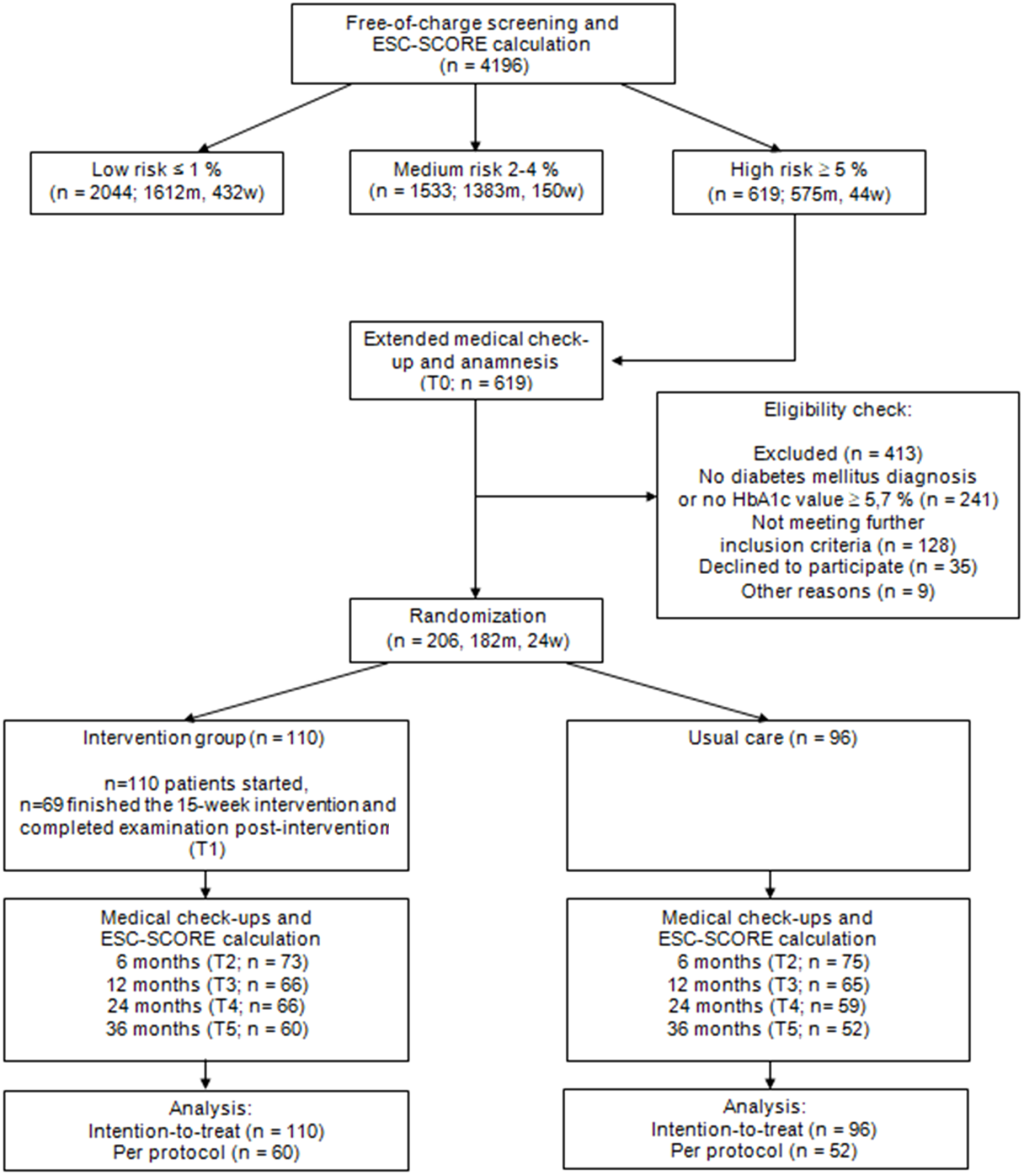
Study flow chart.

The secondary data analysis is reported in accordance with the CONSORT statement (14). Only employees diagnosed with diabetes mellitus (and receiving pharmacological treatment) and/or with glycated hemoglobin (HbA1c) levels ≥ 5.7% were included in this analysis (**Figure 1**). In total, the datasets of n=142 persons with pre-DM (HbA1c levels ≥ 5.7% and < 6.5% without anti-diabetic medication) and n=64 patients with manifest DM (HbA1c levels ≥ 6.5% and/or treated with anti-diabetic medication) were considered. The HbA1c thresholds correspond to the American Diabetes Association cutoffs for the diagnoses of pre-DM and DM (15).

#### 2.2.2 Primary and secondary outcomes

The ESC-SCORE was defined as the primary outcome. It was determined in the INT and CON group at every follow-up examination and thus helped assess the long-term effectiveness of the intervention. The same ESC-SCORE algorithm in its initially published form was used throughout the study (5). The ESC-SCORE provides an accurate prediction of cardiovascular events in Europeans without a history of severe cardiovascular diseases (e.g., coronary heart disease, stroke, peripheral artery disease, heart failure, heart arrhythmia).

To determine the intervention’s direct effectiveness, body weight, body mass index (BMI), waist circumference, glycated hemoglobin (HbA1c), high-sensitive C-reactive protein (hsCRP), total cholesterol, high-density lipoprotein (HDL), low-density lipoprotein (LDL), triglycerides, systolic and diastolic blood pressure and exercise capacity pre- and post-intervention were defined as secondary outcomes.

#### 2.2.3 Statistical analyses

Data are presented as mean values ± standard deviations (SD) and 95% confidence intervals (95%-CI). The “SPSS” program (v. 28.0, IBM Corporation, Armonk, New York, USA) was used for the statistical analyses. Parametric tests were used throughout. When assumptions were violated and when appropriate, non-parametric (rank-based) hypotheses tests were conducted. For baseline comparisons of interval-scaled variables between the two groups, the Student’s t test or the Mann-Whitney U test for unpaired samples were performed. The Chi^2^ test was used to assess differences in the distribution of nominal-scaled variables between the groups. For pre-post-comparisons of interval-scaled variables within the INT group, the Student’s t test or the Wilcoxon signed rank test for paired samples were used. For follow-up analyses within each group (INT and CON), the Friedman test was carried out. To compare changes from baseline between the two groups at the different follow-up time points, the Student’s t test or the Mann-Whitney U test for unpaired samples were used. Data were analyzed intention-to-treat and per protocol. The intention-to-treat cohort included all patients. Missing values in the intention-to-treat analysis were replaced by the last observation carried forward (29.9% missing ESC-SCORE data, 33.2% missing HbA1c data). The per protocol cohort included only those patients who fully adhered to the study protocol. In addition, data from all measurement time points had to be available. Significance was considered at p ≤ 0.05.

#### 2.2.4 Sample size and power calculation

A sample size calculation was performed for the original study *a priori* (4). For this subgroup data analysis, a second power analysis was performed for the ESC-SCORE as the primary outcome *a posteriori* using G Power (v. 3.1.9.7., University of Düsseldorf, Düsseldorf, Germany). For the intention-to-treat analysis, a power of 100% was calculated for the comparison between the ESC-SCORE pre- and post-intervention of the INT group and a power of 94% for the comparison of ESC-SCORE changes from baseline between the INT and CON groups during the follow-up medical check-up 36 months later. For the per protocol analysis, statistical power values of 100% and 83% were calculated, respectively.

## 3 Results

### 3.1 Baseline data

The baseline (T0) data of the subjects of the INT and CON groups are presented in **Table 2**. The ratio of men and women roughly reflects the ratio of employees in the company. The groups were almost perfectly matched for the ESC-SCORE and also did not significantly differ in any other variable, except BMI.

**Table 2:** Study participants´ baseline (T0) characteristics.

Intervention group n=110		Usual care group n=96	p-value
ESC-SCORE result [%]	8.07 ± 5.17 (7.09-9.05)	8.03 ± 4.83 (7.06-9.01)	0.959 ◊
Sex [m/f, n]	96/14	86/10	0.606 †
Age [years]	60.1 ± 8.7 (58.4-61.7)	60.2 ± 7.7 (58.6-61.7)	0.994 ◊
Body weight [kg]	89.6 ± 15.3 (86.7-92.4)	86.4 ± 14.3 (83.5-89.3)	0.076 ◊
BMI [kg/m^2^]	29.62 ± 4.55 (28.76-30.48)	28.19 ± 3.85 (27.41-28.97)	0.019 ◊
Waist circumference [cm]	103.8 ± 10.6 (101.8-105.8) n=109	101.0 ± 12.4 (98.5-103.5) n=95	0.083 #
HbA1c [%]	6.41 ± 0.86 (6.25-6.57)	6.18 ± 0.57 (6.06-6.29)	0.103 ◊
hsCRP [mg/l]	0.31 ± 0.56 (0.20-0.41) n=108	0.33 ± 0.54 (0.22-0.44) n=95	0.752◊
Total cholesterol [mg/dl]	238.9 ± 48.5 (229.7-248.1)	237.4 ± 48.5 (227.6-247.2)	0.908 ◊
HDL [mg/dl]	53.5 ± 12.3 (51.1-55.8)	54.8 ± 12.8 (52.2-57.4)	0.338 ◊
LDL [mg/dl]	150.6 ± 34.4 (144.1-157.1)	149.7 ± 33.6 (142.9-156.5)	0.928 ◊
Triglycerides [mg/dl]	218.0 ± 160.2 (187.7-248.2)	204.5 ± 138.6 (176.5-232.6)	0.982 ◊
Systolic BP [mmHg]	139.8 ± 17.7 (136.4-143.1)	138.1 ± 15.0 (135.1-141.2)	0.417 ◊
Diastolic BP [mmHg]	87.9 ± 11.1 (85.8-90.0)	88.7 ± 9.9 (86.7-90.7)	0.720 ◊
Exercise capacity [W/kg]	1.73 ± 0.47 (1.64-1.82) n=103	1.69 ± 0.43 (1.60-1.78) n=86	0.715 #
Smokers			
Non-smokers	42 (38.2%)	39 (40.6%)	
Current smokers	23 (20.9%)	21 (21.9%)	0.866 †
Ex-smokers	45 (40.9%)	36 (37.5%)	
Anti-diabetic drugs			
Insulin	8 (7.3%)	10 (10.4%)	0.425 †
Oral antidiabetic agents	19 (17.3%)	9 (9.4%)	0.099 †
Other drugs			
ASS	13 (11.8%)	20 (20.8%)	0.078 †
Statins	26 (23.6%)	15 (15.6%)	0.151 †
Anti-hypertensive agents	55 (50.0%)	36 (37.5%)	0.072 †

ESC-SCORE, European Society of Cardiology Systematic Coronary Risk Evaluation; BMI, body mass index; HbA1c, glycated hemoglobin; hsCRP, high-sensitive C-reactive protein; HDL, high-density lipoprotein; LDL, low-density lipoprotein; BP, blood pressure. Means ± standard deviations (SD) and 95% confidence intervals. ◊ Mann-Whitney U test # Student´s t test (unpaired samples) † Chi^2^ test.

### 3.2 Direct effects of the multimodal lifestyle intervention on the ESC-SCORE and important health variables

Pre-post-intervention data (T0-T1) are presented in **Table 3**. The ESC-SCORE decreased significantly, irrespective of the type of analysis conducted (intention-to-treat or per protocol). Nearly all other health-related variables (body weight, BMI, waist circumference, HbA1c, total cholesterol, LDL, triglycerides, systolic and diastolic blood pressure, exercise capacity) also improved significantly. HDL levels remained unchanged and hsCRP levels increased significantly, but very slightly.

**Table 3:** Study participants´ characteristics pre (T0) and post (T1) -intervention.

Intention-to-treat analysis Intervention group Pre-intervention n=110		Intention-to-treat analysis Intervention group Post-intervention n=110	p-value	Per protocol analysis Intervention group Pre-intervention n=69	Per protocol analysis Intervention group Post-intervention n=69	p-value
ESC-SCORE result [%]	8.07 ± 5.17 (7.09-9.05)	6.33 ± 4.31 (5.52-7.15)	<0.001 ○	8.62 ± 5.29 (7.35-9.89)	5.85 ± 3.87 (4.92-6.78)	<0.001 ○
Body weight [kg]	89.6 ± 15.3 (86.7-92.4)	87.8 ± 15.1 (85.0-90.7)	<0.001 ▪	86.8 ± 14.7 (83.2-90.3)	84.0 ± 13.7 (80.7-87.3)	<0.001 ▪
BMI [kg/m^2^]	29.62 ± 4.55 (28.76-30.48)	29.02 ± 4.36 (28.20-29.84)	<0.001 ○	29.08 ± 4.46 (28.01-30.16)	28.13 ± 3.97 (27.17-29.08)	<0.001 ○
Waist circumference [cm]	103.8 ± 10.6 (101.8-105.8) n=109	101.9 ± 10.4 (99.9-103.9) n=109	<0.001 ▪	102.0 ± 10.7 (99.4-104.6) n=68	99.0 ± 9.7 (96.6-101.3) n=68	<0.001 ▪
HbA1c [%]	6.41 ± 0.86 (6.25-6.57)	6.26 ± 0.87 (6.09-6.42)	<0.001 ○	6.29 ± 0.80 (6.09-6.48)	6.04 ± 0.75 (5.86-6.22)	<0.001 ○
hsCRP [mg/l]	0.31 ± 0.56 (0.20-0.41) n=108	0.32 ± 0.64 (0.20-0.41) n=108	0.035 ○	0.24 ± 0.33 (0.16-0.32) n=67	0.26 ± 0.52 (0.14-0.39) n=67	0.035 ○
Total cholesterol [mg/dl]	238.9 ± 48.5 (229.7-248.1)	219.7 ± 49.5 (210.3-229.0)	<0.001 ○	232.2 ± 44.2 (221.6-242.9)	201.5 ± 36.6 (192.8-210.3)	<0.001 ○
HDL [mg/dl]	53.5 ± 12.3 (51.1-55.8)	53.8 ± 13.0 (51.3-56.2)	0.625 ○	54.8 ± 12.8 (51.7-57.9)	55.3 ± 13.8 (52.0-58.6)	0.625 ○
LDL [mg/dl]	150.6 ± 34.4 (144.1-157.1)	134.3 ± 34.3 (127.9-140.8)	<0.001 ○	147.6 ± 33.6 (139.5-155.6)	121.6 ± 26.4 (115.3-128.0)	<0.001 ○
Triglycerides [mg/dl]	218.0 ± 160.2 (187.7-248.2)	188.6 ± 149.5 (160.3-216.8)	<0.001 ○	191.2 ± 130.2 (159.9-222.4)	144.3 ± 90.7 (122.5-166.1)	<0.001 ○
Systolic BP [mmHg]	139.8 ± 17.7 (136.4-143.1)	132.7 ± 13.9 (130.1-135.3)	<0.001 ▪	142.5 ± 19.2 (137.8-147.1)	131.2 ± 13.6 (127.9-134.5)	<0.001 ▪
Diastolic BP [mmHg]	87.9 ± 11.1 (85.8-90.0)	84.5 ± 9.6 (82.7-86.3)	<0.001 ○	88.5 ± 11.7 (85.7-91.3)	83.1 ± 9.1 (80.9-85.3)	<0.001 ▪
Exercise capacity [W/kg]	1.73 ± 0.47 (1.64-1.82) n=103	1.89 ± 0.50 (1.80-1.99) n=103	<0.001 ○	1.78 ± 0.51 (1.66-1.91) n=65	2.04 ± 0.50 (1.92-2.17) n=65	<0.001 ▪

ESC-SCORE, European Society of Cardiology Systematic Coronary Risk Evaluation; BMI, body mass index; HbA1c, glycated hemoglobin; hsCRP, high-sensitive C-reactive protein; HDL, high-density lipoprotein; LDL, low-density lipoprotein; BP, blood pressure. Means ± standard deviations (SD) and 95% confidence intervals. ○ Wilcoxon signed rank test ▪ Student´s t test (paired samples).

### 3.3 Follow-up and long-term effects of the multimodal lifestyle intervention on the ESC- SCORE

**Figure 2:**
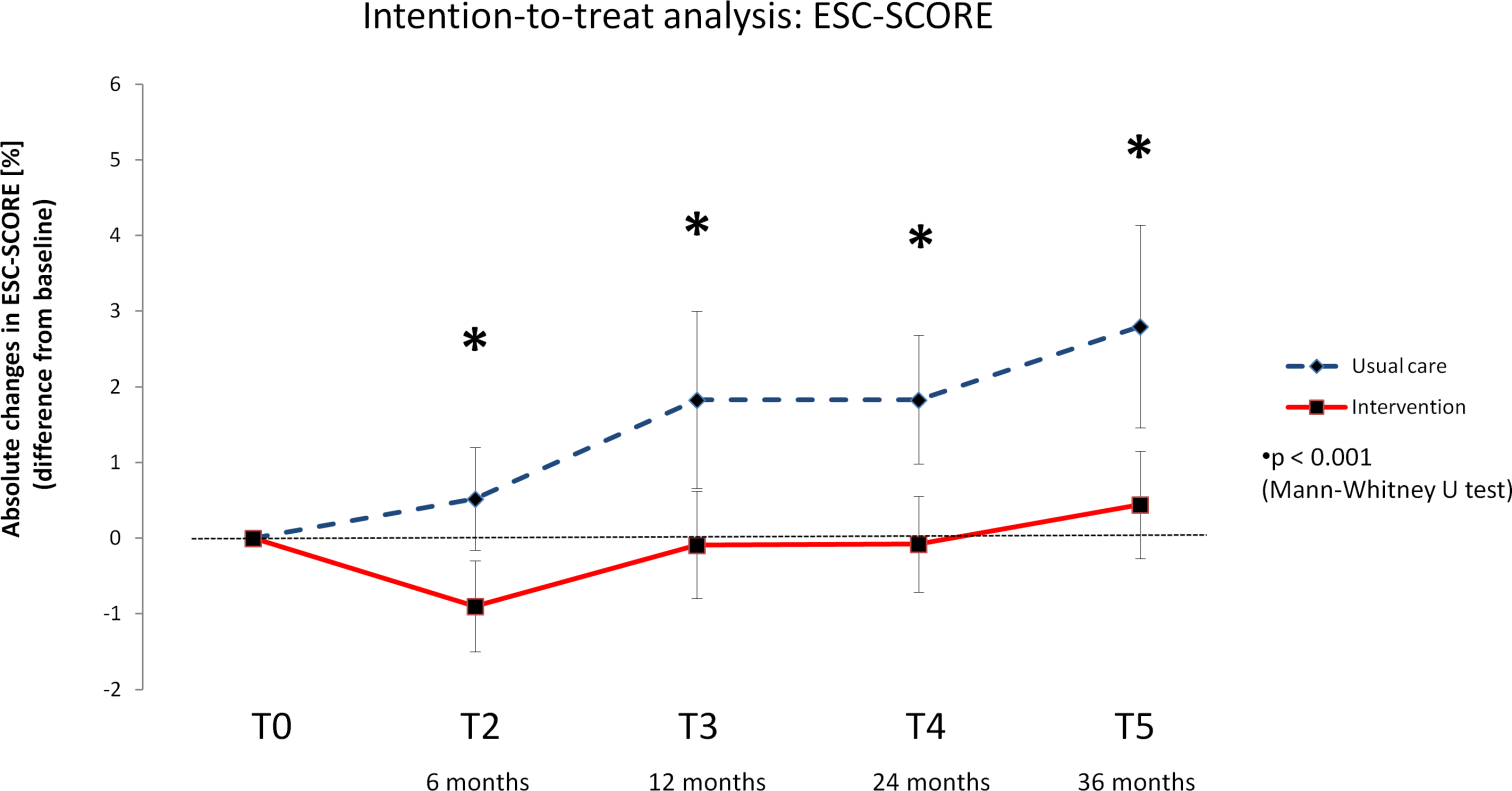
Delta values of the European Society of Cardiology – Systematic Coronary Risk Evaluation score (ESC-SCORE) – Intention-to-treat analysis. Means with 95% confidence intervals.

**Figure 3:**
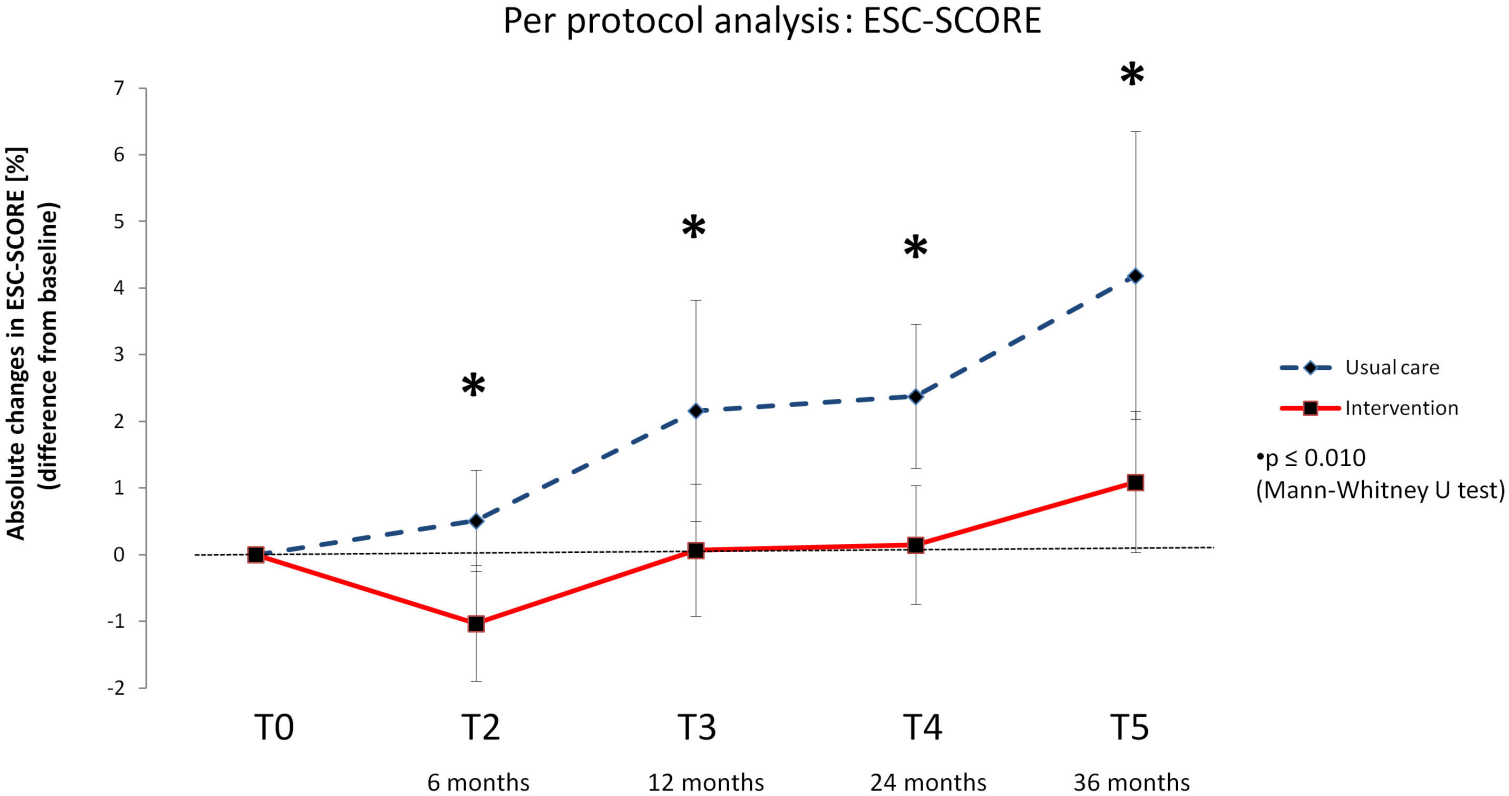
Delta values of the European Society of Cardiology – Systematic Coronary Risk Evaluation score (ESC-SCORE) –Per protocol analysis. Means with 95% confidence intervals.

There was a significant overall time effect for the ESC-SCORE in each group (INT and CON) from T0 across all follow-up time points (T2, T3, T4, T5) (Friedman test: p<0.001), which was evident in both the intention-to-treat and per protocol analyses (**Supplemental Data File: ESM 1**). It appears that the ESC-SCORE of the INT group increased only very slightly in the long term after the intervention, while it increased more in the CON group. This is reflected in the changes from baseline. The delta values differed significantly between the groups (INT and CON) at each time point (T2, T3, T4, T5), with the intervention group achieving more favorable results in the intention-to-treat (**Figure 2**) as well as the per protocol analysis (**Figure 3**).

To clarify whether there is a difference in the primary outcome between pre-DM and DM patients, a further subgroup analysis was performed for ESC-SCORE changes (**Supplemental Data File: ESM 2** **).** The intention-to-treat analysis revealed that the pre-DM patients’ (INT: n=72, CON: n=70) results were quite similar to those of all patients (pre-DM/DM patients). Delta values differed significantly between the groups (INT and CON) at each time point (T2, T3, T4, T5), with the intervention group achieving more favorable results. In DM patients (INT: n=38, CON: n=26), a significant difference in ESC-SCORE changes was evident after the lifestyle intervention, with better results in the INT group. However, from T3 onward, there was no longer a significant difference in delta values between the groups (INT and CON). It should be noted that ESC-SCORE baseline values were significantly higher in pre-DM than in DM patients in both groups (INT: pre-DM: 9.16 ± 4.99% (95%-CI: 7.99-10.33%), DM: 6.00 ± 4.95% (95%-CI: 4.38-7.63%), U test: p<0.001; CON: pre-DM: 8.54 ± 4.12% (95%-CI: 7.55-9.52%), DM: 6.67 ± 6.24% (95%-CI: 4.15-9.20%), U test: p=0.017). Due to the small number of included DM patients (INT: n=15, CON: n=12), no subgroup analysis was performed in the per protocol cohort.

### 3.4 Follow-up and long-term effects of the multimodal lifestyle intervention on glycemic control

There was a significant overall time effect for the HbA1c levels in the INT group from T0 across all follow-up time points (T2,T3,T4,T5) (Friedman test: p<0.001), which was evident in both the intention-to-treat and per protocol analyses (**Supplemental Data File: ESM 3**). There were no significant HbA1c changes in the CON group. Of all pre-DM patients from the per protocol cohort, 5% developed manifest DM in the INT and 22% in the CON group (from T0 to T5). Half of them started treatment with anti-diabetic medication.

### 3.5 Adverse events during the intervention

There were no adverse events during the intervention.

## 4 Discussion

DM can drastically increase the risk of CVDs. The INTERHEART study, which collected data from more than 27,000 subjects in 52 countries, identified DM as a strong risk factor for acute myocardial infarction (9). Other famous large-scale studies such as the Framingham study or the San Antonio Heart Study found increased CVD mortality rates in DM patients compared with non-diabetic subjects from the general population (7, 8). Furthermore, the understanding of the pathogenesis of CVDs in the context of DM improves continuously, with hyperglycemia, hyperinsulinemia and hypercoagulability playing important roles in increased CVD risk and mortality (16, 17). Lifestyle interventions that can prevent the development of CVDs or that have a positive effect on their progression should therefore be strongly recommended as preventive measures not only for patients with manifest DM, but also for those with pre-DM (18, 19).

The secondary data analysis of the PreFord study shows that the cardiovascular risk of persons with pre-DM/DM can be substantially reduced through the multimodal lifestyle program applied in the study. There was a direct effect on several health variables and the ESC-SCORE after 15 weeks. Over the next 3 years of follow-up, there were more favorable results in the INT group.

The ESC-SCORE reflects the probability of dying in the next 10 years from a cardiovascular event (5). The ESC-SCORE used in this study is calculated based on age, systolic blood pressure, smoking habits and total cholesterol values (5, 6). Although the algorithm does not consider pre-DM or diabetes status, the ESC-SCORE is nonetheless suitable for a rough assessment of the cardiovascular risk in the subgroup studied, because the relationship of the other risk factors with CVDs are almost parallel in individuals with and without DM (5, 20). However, the risk of persons with DM is generally higher. According to the ESC- SCORE’s instructions, it should be considered for the interpretation that the calculated risk at every risk factor combination can be at least twice as high in men and up to 4-fold higher in women with manifest DM (5). It must therefore be assumed that the actual CVD risk tends to be underestimated by the ESC-SCORE value for the subgroup studied, but because many more pre-DM patients than patients with manifest DM were included in the analysis, the underestimation should not be too far-reaching.

The overall results suggest a clear positive health effect of the intervention for the subgroup studied, which is very similar to the effect for the entire study cohort group (4). Persons in the intervention group generally benefited from the multimodal lifestyle program, which was reflected in more favorable ESC-SCORE changes compared to those in the usual care control group over the course of the study. Multimodal interventions that also target self-empowerment, such as the program in the PreFord study, promise long-term effectiveness, which in turn may also be cost-effective (21). Kähm et al. (22) estimated the costs for diabetic complications in German patients. End-stage renal disease, amputations, stroke, myocardial infarction and ischemic heart disease were deemed very cost-intensive. Indirect costs related to lost productivity and work ability due to diabetes and its complications are also very high (23). Magliano et al. (24) demonstrated that “productivity-adjusted life years” were reduced by 11.6% and 10.5% among men and women with DM, respectively. Interventions that focus on persons with pre-DM and DM and which are initiated early in working life could thus help reduce work absenteeism and protect the workforce by preventing the development of disease complications.

A closer look at the long-term effects on the ESC-SCORE changes (intention-to-treat analysis) implies that pre-DM patients in particular benefited from the lifestyle intervention. Further measures may be necessary to achieve more beneficial effects in patients with manifest DM. However, it should be noted that the pre-DM patients already had higher values at the beginning of the study, so that possible improvements may be more pronounced in them than in the DM patients. However, the result should not be overestimated, as only 42% of the DM patients of the intention-to-treat analysis fully adhered to the study protocol.

The strategy for raising awareness of CVD risk at the workplace through flyers and offering a quick medical check-up free of charge could—as demonstrated in the present study—motivate workers with pre-DM and DM to participate in multimodal therapy. Despite the noted drop-out rate during the intervention of 37% among those with pre-DM and DM (for all study participants, the rate was 32%), the intention-to-treat analysis nevertheless indicated significant and clinically meaningful improvements post-intervention, underscoring the program’s overall efficacy.

The PreFord study has some limitations, which have already been pointed out in the initial publication (4). One limitation, for example, is the fact that there could be concerns against the employer who pushed the study, so that some employees did not participate in the CVD mortality risk screening due to concerns that their health data could be misused. Therefore, the representativeness of the results for the entire company cannot be guaranteed. Another limitation is that very few women were included. Therefore, the question is to what extent the results are gender-specific. This cannot be clarified based on the present data.

An additional point that might be of interest, especially for the secondary analysis, is that no distinction was made between the types of DM. Among the 13 insulin-dependent patients, some patients with type 1 DM may have been included. However, Juutilainen et al. (25) showed in an 18-year observational study that there was no major difference between middle-aged individuals with T1DM and T2DM in terms of their CVD mortality risk (onset of the disease was > 30 years in both groups). However, other data suggest a greater mortality risk for T2DM patients compared with T1DM patients when the age of onset of the diabetic disease is earlier in both groups (15-30 years) (26).

Furthermore, there was a minor, but statistically significant difference in BMI values between the INT and CON group, which might have affected the development of health values. However, for the primary outcome (ESC-SCORE), the groups were almost perfectly matched.

## 5 Conclusion

In conclusion, attracting company employees who are at high CVD mortality risk to participate in a multimodal lifestyle program following a free CVD mortality risk screening at their workplace may be a successful strategy for CVD prevention, particularly in patients with pre-DM/DM. The multimodal intervention used in the PreFord study was suitable for improving the health of company employees with pre-DM/DM and for reducing their CVD mortality risk in the long term.

## Data availability statement

The original contributions presented in the study are included in the article/**Supplementary Material**. Further inquiries can be directed to the corresponding author.

## Ethics statement

The studies involving human participants were reviewed and approved by the Ethics Committee of the University of Cologne. The patients/participants provided their written informed consent to participate in this study.

## Author contributions

CB had the idea for this paper. CB and HH performed the statistical analyses. CB wrote and revised the manuscript. All other authors (D-BG, CA, SM, BB-W, JL, GH, KW, CH, MS, H-GP) have contributed substantially to the design, acquisition, analysis and interpretation of study data from the PreFord study and gave their intellectual input to the present manuscript. All authors contributed to the article and approved the submitted version.
