# Supplementary material for: Lifestyle intervention reduces risk score for cardiovascular mortality in company employees with pre-diabetes or diabetes mellitus – A secondary analysis of the PreFord randomized controlled trial with 3 years of follow-up
Source: Front Endocrinol (Lausanne). 2023 Feb 23;14:1106334. doi: 10.3389/fendo.2023.1106334 (PMC9992873; doi:10.3389/fendo.2023.1106334)
Supplement: Supplementary file 1 [file DataSheet_1.doc]

**Supplemental Data File 1 (ESM 1).** Systematic Coronary Risk Evaluation score (ESC-SCORE) results.

**Table S1. Intention- to-treat analysis: ESC-SCORE data.**

|  | Intervention n=110 Ŧ | Control n=96 Ŧ |
| --- | --- | --- |
| T0 [%] | 8.07 ± 5.17 (7.09-9.05) | 8.03 ± 4.82 (7.06-9.01) |
| T2 [%] | 7.18 ± 4.56 (6.32-8.04) | 8.56 ± 5.31 (7.48-9.63) |
| T3 [%] | 7.99 ± 5.32 (6.98-8.99) | 9.87 ± 7.85 (8.28-11.46) |
| T4 [%] | 7.99 ± 4.97 (7.05-8.93) | 9.86 ± 6.30 (8.58-11.14) |
| T5 [%] | 8.51 ± 5.70 (7.43-9.59) | 10.83 ± 8.80 (9.05-12.62) |

ESC-SCORE: European Society of Cardiology Systematic Coronary Risk Evaluation . Means ± standard deviations (SD) and 95% confidence intervals. Ŧ Significant time effect in both groups. Friedman test Intervention: p<0.001 (time effect), Friedman test Control: p<0.001 (time effect).

**Table S2. Per protocol analysis: ESC-SCORE data.**

|  | Intervention n=60 Ŧ | Control n=52 Ŧ |
| --- | --- | --- |
| T0 [%] | 8.21 ± 4.95 (6.93-9.49) | 7.23 ± 4.12 (6.08-8.38) |
| T2 [%] | 7.12 ± 4.56 (6.01-8.36) | 7.75 ± 4.67 (6.44-9.05) |
| T3 [%] | 8.28 ± 5.71 (6.80-9.75) | 9.39 ± 8.44 (7.04-11.74) |
| T4 [%] | 8.36 ± 5.26 (7.00-9.72) | 9.61 ± 6.22 (7.88-11.34) |
| T5 [%] | 9.30 ± 6.40 (7.65-10.95) | 11.42 ± 10.40 (8.52-14.31) |

ESC-SCORE: European Society of Cardiology Systematic Coronary Risk Evaluation . Means ± standard deviations (SD) and 95% confidence intervals. Ŧ Significant time effect in both groups. Friedman test Intervention: p<0.001 (time effect), Friedman test Control: p<0.001 (time effect).
