# Supplementary material for: Lifestyle intervention reduces risk score for cardiovascular mortality in company employees with pre-diabetes or diabetes mellitus – A secondary analysis of the PreFord randomized controlled trial with 3 years of follow-up
Source: Front Endocrinol (Lausanne). 2023 Feb 23;14:1106334. doi: 10.3389/fendo.2023.1106334 (PMC9992873; doi:10.3389/fendo.2023.1106334)
Supplement: Supplementary file 2 [file DataSheet_2.doc]

**Supplemental Data File 2 (ESM 2).** Delta values of the European Society of Cardiology – Systematic Coronary Risk Evaluation score (ESC-SCORE) – Intention-to-treat subgroup analysis in company employees with pre-diabetes (Figure S1) and diabetes mellitus (Figure S2).


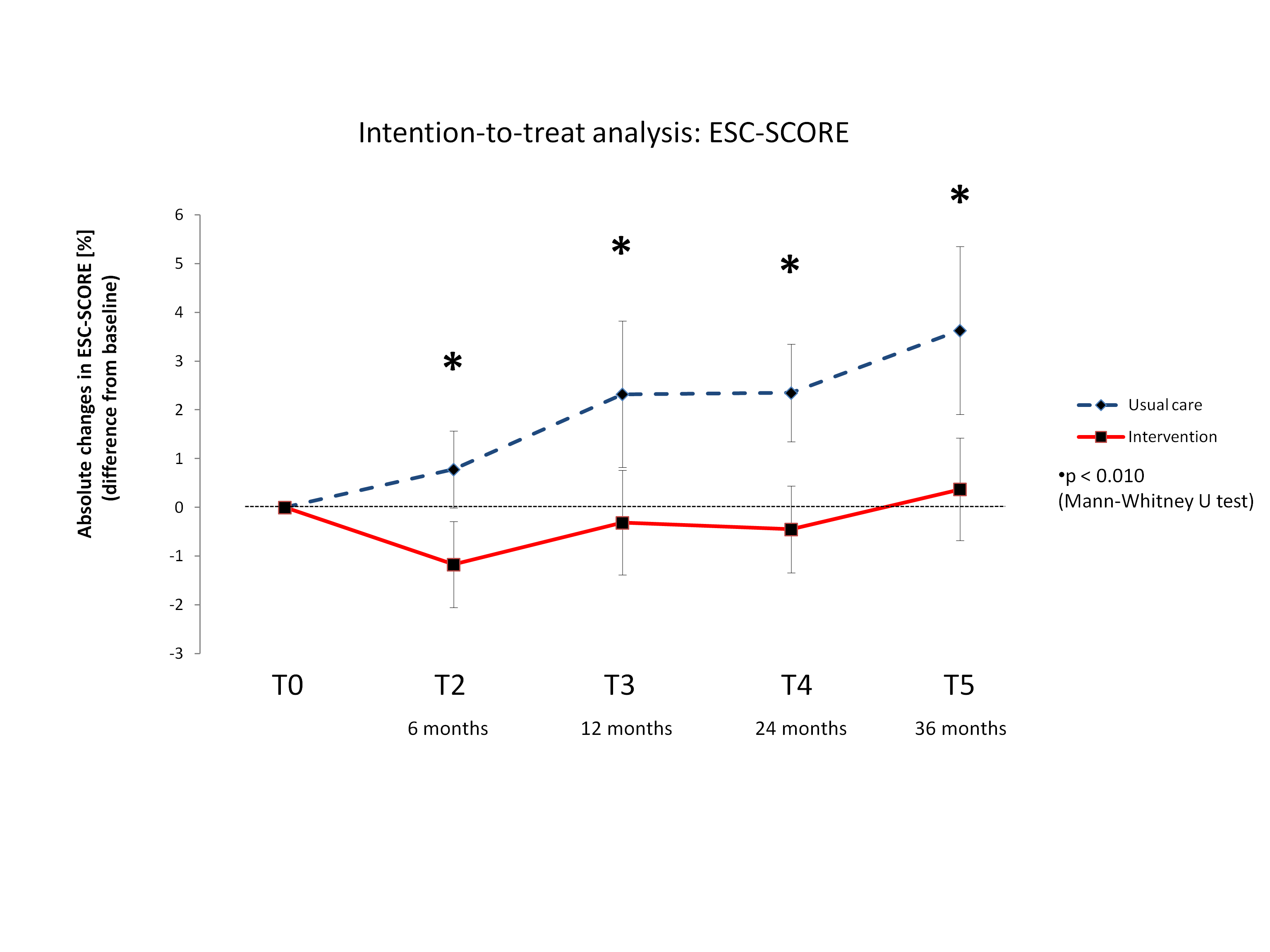


**Figure S1.** Delta values of the European Society of Cardiology – Systematic Coronary Risk Evaluation score (ESC-SCORE) – Intention-to-treat subgroup analysis in company employees with pre-diabetes. Means with 95% confidence intervals.


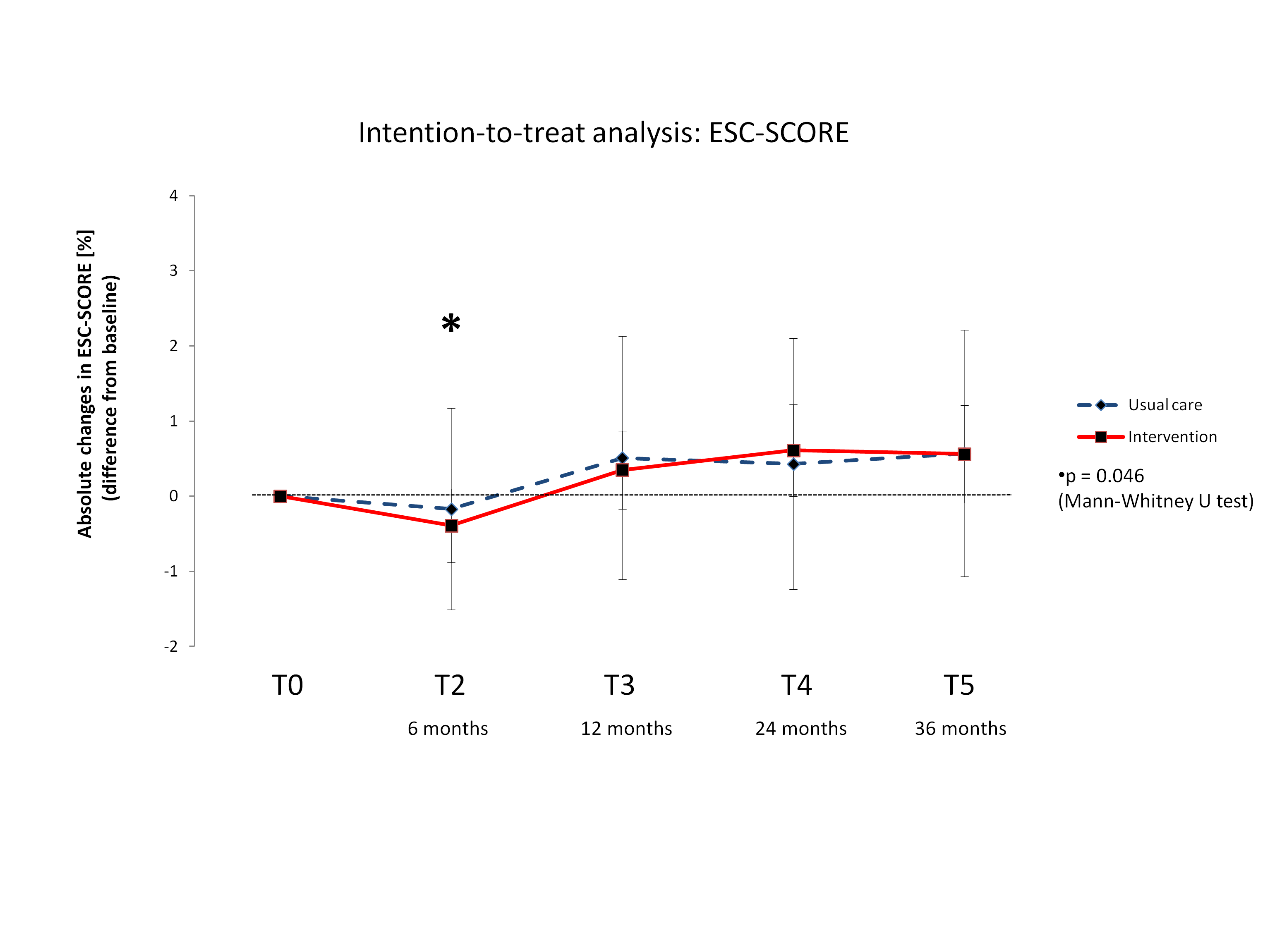


**Figure S2.** Delta values of the European Society of Cardiology – Systematic Coronary Risk Evaluation score (ESC-SCORE) – Intention-to-treat subgroup analysis in company employees with diabetes mellitus. Means with 95% confidence intervals.
