# Supplementary material for: Lifestyle intervention reduces risk score for cardiovascular mortality in company employees with pre-diabetes or diabetes mellitus – A secondary analysis of the PreFord randomized controlled trial with 3 years of follow-up
Source: Front Endocrinol (Lausanne). 2023 Feb 23;14:1106334. doi: 10.3389/fendo.2023.1106334 (PMC9992873; doi:10.3389/fendo.2023.1106334)
Supplement: Supplementary file 3 [file DataSheet_3.doc]

**Supplemental Data File 3 (ESM 3).** Glycated hemoglobin (HbA1c) levels.

**Table S3. Intention- to-treat analysis: HbA1c data.**

|  | Intervention n=110 Ŧ | Control n=96 |
| --- | --- | --- |
| T0 [%] | 6.41 ± 0.86 (6.25-6.57) | 6.18 ± 0.57 (6.06-6.29) |
| T2 [%] | 6.27 ± 0.83 (6.11-6.43) | 6.22 ± 0.64 (6.09-6.35) |
| T3 [%] | 6.39 ± 0.85 (6.23-6.55) | 6.26 ± 0.62 (6.14-6.38) |
| T4 [%] | 6.39 ± 0.83 (6.23-6.55) | 6.27 ± 0.65 (6.14-6.40) |
| T5 [%] | 6.40 ± 0.86 (6.24-6.57) | 6.20 ± 0.62 (6.08-6.33) |

HbA1c: glycated hemoglobin. Means ± standard deviations (SD) and 95% confidence intervals. Ŧ Significant time effect. Friedman test Intervention: p<0.001 (time effect), Friedman test Control: p=0.110 (no time effect).

**Table S4. Per protocol analysis: HbA1c data.**

|  | Intervention n=49 Ŧ | Control n=38 |
| --- | --- | --- |
| T0 [%] | 6.14 ± 0.53 (5.99-6.30) | 6.13 ± 0.56 (5.95-6.32) |
| T2 [%] | 5.96 ± 0.51 (5.81-6.10) | 6.25 ± 0.73 (6.01-6.49) |
| T3 [%] | 6.16 ± 0.56 (6.00-6.32) | 6.29 ± 0.67 (6.07-6.51) |
| T4 [%] | 6.17 ± 0.60 (5.99-6.34) | 6.29 ± 0.80 (6.03-6.55) |
| T5 [%] | 6.18 ± 0.61 (6.00-6.36) | 6.22 ± 0.77 (5.97-6.48) |

HbA1c: glycated hemoglobin. Means ± standard deviations (SD) and 95% confidence intervals. Ŧ Significant time effect. Friedman test Intervention: p<0.001 (time effect), Friedman test Control: p=0.408 (no time effect).
